# Gene Expression Levels of the Prolyl Hydroxylase Domain Proteins *PHD1* and *PHD2* but Not *PHD3* Are Decreased in Primary Tumours and Correlate with Poor Prognosis of Patients with Surgically Resected Non-Small-Cell Lung Cancer

**DOI:** 10.3390/cancers13102309

**Published:** 2021-05-12

**Authors:** Ana Koren, Matija Rijavec, Tomaž Krumpestar, Izidor Kern, Aleksander Sadikov, Tanja Čufer, Peter Korošec

**Affiliations:** 1 University Clinic of Respiratory and Allergic Diseases Golnik, 4204 Golnik, Slovenia; matija.rijavec@klinika-golnik.si (M.R.); tomaz.krumpestar@gmail.com (T.K.); izidor.kern@klinika-golnik.si (I.K.); tanja.cufer@mf.uni-lj.si (T.Č.); peter.korosec@klinika-golnik.si (P.K.); 2 Faculty of Computer and Information Science, University of Ljubljana, 1000 Ljubljana, Slovenia; aleksander.sadikov@fri.uni-lj.si; 3 Medical Faculty, University of Ljubljana, 1000 Ljubljana, Slovenia

**Keywords:** non-small-cell lung cancer, prolyl hydroxylase domain proteins, mRNA expression, prognosis

## Abstract

**Simple Summary:**

Hypoxia correlates with poor prognosis in several cancer types, including lung cancer. Prolyl hydroxylase domain proteins (PHDs) belong to an evolutionarily conserved superfamily of dioxygenases that play a role in cell oxygen sensing and homeostasis. In this study, we evaluated *PHD1*, *PHD2* and *PHD3* mRNA expression in 60 NSCLC tumours and compared it to that in normal lungs and evaluated the prognostic significance of these differences for distinguishing the survival of NSCLC patients treated with radical surgery. Our results showed that the mRNA expression *PHD1* and *PHD2* in NSCLC primary tumours was decreased, which correlated with larger tumour size and poor prognosis of patients. *PHD1* also showed borderline independent prognostic value in multivariate analysis. In contrast, we found no associations between *PHD3* expression and any of the observed parameters. Our results suggest that loss of *PHD1* and *PHD2* expression is associated with the development and progression of NSCLC, whereas *PHD1* could be further assessed as a prognostic marker in NSCLC.

**Abstract:**

Background: Hypoxia correlates with poor prognosis in several cancer types, including lung cancer. Prolyl hydroxylase domain proteins (PHDs) play a role in cell oxygen sensing, negatively regulating the hypoxia-inducible factor (HIF) pathway. Our study aim was to evaluate *PHD1*, *PHD2* and *PHD3* mRNA expression levels in primary tumours and normal lungs of non-small-cell lung cancer (NSCLC) patients and to correlate it with selected regulators of HIF signalling, with clinicopathological characteristics and overall survival (OS). Methods: Tumour tissue samples were obtained from 60 patients with surgically resected NSCLC who were treated with radical surgery. In 22 out of 60 cases, matching morphologically normal lung tissue was obtained. *PHD1*, *PHD2* and *PHD3* mRNA expressions were measured using RT-qPCR. Results: The *PHD1* and *PHD2* mRNA levels in primary tumours were significantly decreased compared to those in normal lungs (both *p* < 0.0001). *PHD1* and *PHD2* expression in tumours was positively correlated (*r_s_* = 0.82; *p* < 0.0001) and correlated well with HIF pathway downstream genes *HIF1A*, *PKM2* and *PDK1*. Decreased *PHD1* and *PHD2* were associated with larger tumour size, higher tumour stage (*PHD1* only) and squamous cell carcinoma. Patients with low *PHD1* and patients with low *PHD2* expression had shorter OS than patients with high *PHD1* (*p* = 0.02) and *PHD2* expression (*p* = 0.01). *PHD1* showed borderline independent prognostic values in multivariate analysis (*p* = 0.06). In contrast, we found no associations between *PHD3* expression and any of the observed parameters. Conclusions: Our results show that reduced expression of *PHD1* and *PHD2* is associated with the development and progression of NSCLC. *PHD1* could be further assessed as a prognostic marker in NSCLC.

## 1. Introduction

Tumour hypoxia is associated with an aggressive tumour phenotype and metastasis [1,2] and is a known source of treatment resistance and poor survival in solid malignancies, including lung cancer [3,4]. Lung cancer is the most commonly diagnosed cancer (11.6% of total cases) and the main cause of cancer-related mortality (18.4% of total cancer deaths) worldwide [5]. Non-small-cell lung cancer (NSCLC) represents up to 90% of lung cancer cases, with a further increase in its proportion within all lung cancer cases expected in the future [6]. NSCLC represents one among the foremost biologically various cancers, and studies on NSCLC have led to the acknowledgement of multiple clinically important genetic subtypes [7,8]. Despite important enhancements in designation and treatment, approximately two-thirds of patients with lung cancer have locally advanced or metastatic disease at the time of diagnosis, and also the prognosis patients remains poor, with the worldwide overall 5-year survival being only 10–20% [9]. Prognostic staging of lung cancer now includes both anatomic factors (TNM classification) and tumour-specific biological markers [10,11].

Prolyl hydroxylase domain proteins (PHDs) belong to an evolutionarily conserved superfamily of dioxygenases that play a role in cell oxygen sensing and homeostasis [12]. The family comprises at least three prolyl hydroxylase isoenzymes, PHD1, PHD2 and PHD3 [13]. PHDs play an important role in the regulation of the hypoxia-inducible factor (HIF) pathway, hydroxylating HIF in normal oxygen conditions, which leads to proteasome-mediated degradation of HIF proteins. Under hypoxic conditions, the hydroxylase activity of the PHD enzymes is inhibited, leading to stabilization of HIF and activation of target genes [14]. HIF1α isoform, in particular, regulates the metabolism of glucose, promoting glycolysis and inhibiting oxygen-dependent metabolism of glucose [15]. Due to their performance as inhibitors of HIF stability, PHDs have been projected to function as tumour suppressors in several cancer types [16,17]. Lending support to this theory, decreased PHD2 expression in cancer cells was associated with faster tumour growth and angiogenesis [18], whereas increased expression of PHD1 inhibited tumour growth [19] in mouse models of colon cancer.

The data regarding the expression of PHDs in lung cancer are scarce and contradictory. The results of a recent study performed in cellular models of lung cancer have shown that the establishment of hypoxia in NSCLC cell lines resulted in a significant decrease in PHD3 protein expression, whereas inhibition of PHD3 resulted in enhanced viability, migration and invasion potential of cancer cells [20]. Giatromanolaki et al. [21] studied the expression of PHDs in lung cancer with immunohistochemical (IHC) staining of 73 NSCLC primary tumour and 10 normal lung samples. All three PHD isoforms were strongly expressed in normal lungs, whereas 24 (32.9%), 33 (45.2%), and 36 (49.3%) NSCLC samples had high expression of PHD1, PHD2, and PHD3, respectively. Additionally, the FIH (factor-inhibiting HIF) group of enzymes was largely coexpressed with PHDs. Chen et al. examined *PHD* mRNA expression by quantitative reverse transcription PCR (RT-qPCR) in 62 paired normal and NSCLC samples. The results of this study indicated that the mRNA expression of PHDs, especially *PHD3*, was much higher in lung cancer tissue than in adjacent normal tissue; increased expression of *PHD1*, *PHD2* and *PHD3* occurred in 53 (85.5%), 48 (77.4%), and 59 (95.2%) NSCLC patient samples, respectively, compared with paired adjacent normal tissue samples. Conversely, in this study, low *PHD3* was associated with higher clinical stage and poor differentiation of tumours [22]. Considering these data, PHD expression in lung cancer has not yet been fully explained and deserves further evaluation.

The aim of our study was to evaluate *PHD1*, *PHD2* and *PHD3* mRNA expression levels in primary tumours and normal lung tissue of surgically resected NSCLC patients and to correlate their expression with selected regulators of HIF signalling and with clinicopathological characteristics and overall survival of NSCLC patients who were treated with radical surgery.

## 2. Materials and Methods

### 2.1. Study Population and Collection of Samples

Tumour tissue samples were obtained from 60 consecutive patients with surgically resected NSCLC who were treated in a routine clinical setting at the University Clinic of Respiratory and Allergic Diseases Golnik from 2012 to 2014. All patients received radical surgery, while neoadjuvant and adjuvant platinum-based chemotherapy was performed according to the guidelines valid at that time [23]. In 22 out of 60 cases, matching morphologically normal lung tissue was obtained. None of the patients had any other malignancies. All patients had pathologically confirmed NSCLC and were diagnosed, treated, and followed by standard clinical practice at University Clinic Golnik. Tissue samples were collected immediately after surgery by an experienced pathologist and histologic diagnosis was decided based on microscopic characteristics according to the WHO classification. Normal lung tissue was sampled in the same resected lobe as far as possible from the primary tumour. The noninvolvement of cancer cells in normal tissue samples was confirmed histologically after hematoxylin and eosin staining. All tissue samples were stored in RNAlater (Qiagen GmbH, Hilden, Germany) at –40 °C till RNA isolation. This study was approved by the Slovenian National Committee for Medical Ethics (protocol N° 40/04/12). Written informed consent was obtained from each participant before being included in the study.

### 2.2. RNA Isolation and cDNA Synthesis

Total RNA from tumour and normal lung tissue samples and A549 cells was isolated miRNeasy Mini Kit (Qiagen) in line with the manufacturer’s instructions. All RNA samples were treated with RNase-free DNase (Qiagen) and reverse transcribed to cDNA with high-capacity cDNA Reverse Transcription Kit (Applied Biosystems, Foster City, CA, USA) as previously described [23,24,25].

### 2.3. RT-qPCR

cDNA was quantified with RT-qPCR (ABI PRISM 7500 Real-Time PCR System; Applied Biosystems) under standard conditions using TaqMan Fast Advanced Master Mix (Applied Biosystems). We utilized TaqMan EGLN2 (Hs01091275_m1), EGLN1 (Hs00254392_m1), and EGLN3 (Hs00222966_m1) assays to quantify *PHD1*, *PHD2* and *PHD3* mRNA expression in primary tumours and normal lungs. TaqMan HIF1A (Hs00153153_m1), PKM2 (Hs00987255_m1) and PDK1 (Hs00176853_m1) were used for quantification of *HIF1A*, *PKM2* and *PDK1* in primary tumours. All measurements were done in triplicate for each sample, and relative gene expression was analysed using the ΔΔCt method [26]. Through this method, the amounts of target gene mRNA were normalized to an endogenous control and related to a calibrator sample using the formula RQ (sample) = 2^−(ΔCt sample−ΔCt calibrator)^. Glyceraldehyde-3-phosphate dehydrogenase (GAPDH; 4333764) was used as the endogenous control (Applied Biosystems). All samples with threshold cycles ≥ 38.0 were considered negative. A549 cells were used as calibrator.

### 2.4. A549 Cell Culture

Human lung adenocarcinoma (AC)-derived cell line A549 was cultured at 5% CO_2_ and 37 °C in RPMI-1640 medium supplemented with 10% FBS, 2.5 mM L-glutamine and 1% penicillin-streptomycin (all Sigma, St. Louis, MO, USA). Cells were originally bought from the European Collection of Authenticated Cell Cultures (ECACC; Public Health England, Salisbury, UK) and were verified by STR fingerprinting. For RNA harvest, cells were grown to confluence, washed with PBS and then RNA was extracted as described in Section 2.2.

### 2.5. Statistics

Comparisons of *PHD1*, *PHD2* and *PHD3* mRNA expression levels between groups (tumours vs. normal lungs) were evaluated with the Mann–Whitney U test or the Wilcoxon test, as appropriate. Spearman’s rank correlation coefficient analysis was performed to determine associations between mRNA expressions. The relationship between the mRNA expression levels of the *PHD1*, *PHD2* and *PHD3* genes and patient characteristics was evaluated using the Mann–Whitney test. Overall survival (OS) was defined as the period of time in months from the date of diagnosis to the date of death (event) or last follow-up (censored data). In the absence of any meaningful or predefined cut-offs, the optimal cut-off value between low and high PHD mRNA expression levels for this cohort of patients was set at the lower quartile (Q1). OS was estimated by the Kaplan–Meier methodology, and the log-rank test was used to compare different categories. The independent prognostic value of each continuously distributed individual marker was tested in a Cox proportional hazards regression model. All variables with *p* ≤ 0.250 in univariate analysis were considered for and included in the multivariate analysis. A *p*-value below 0.05 was considered statistically significant. All reported *p*-values are two-tailed. All statistical analyses were carried out using SPSS (version 21, Chicago, IL, USA) and GraphPad Prism software (version 8, San Diego, CA, USA).

## 3. Results

### 3.1. Patient Characteristics

The clinicopathological characteristics of the 60 NSCLC patients included in the study are presented in Table 1. Thirty-three out of 60 patients (55.0%) were male and the median age was 61 years (range 42–79 years). The vast majority of patients were current or former smokers (51/60; 85.0%) and had good performance status (PS) (PS < 2; 58/60; 96.7%). Of the 60 patients, 35 (58.4%) had adenocarcinoma, and 20 (33.3%) had squamous cell carcinoma. All patients had limited disease (31/60, 51% pTNM stage I; 16/60, 27% pTNM stage II, 13/60, 22% pTNM stage III) [25] and were treated with radical surgery. Four out of 60 patients received neoadjuvant platinum-based chemotherapy before surgery. These individuals were clinically stage III patients with potentially resectable N2 disease. The remaining pTNM stage III patients who did not receive neoadjuvant chemotherapy were diagnosed as resectable single-station N2 disease or were clinically diagnosed as stage I or stage II disease. Twenty-three out of 60 patients also received adjuvant platinum-based chemotherapy after surgery.

### 3.2. PHD mRNA Levels in NSCLC Primary Tumours and Normal Lungs

**Figure 1:**
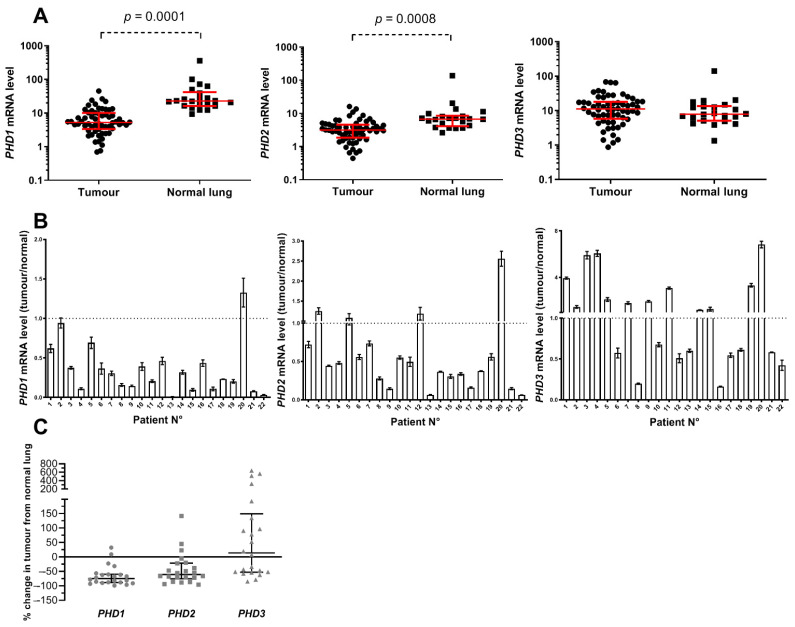
mRNA expression of *PHD1*, *PHD2* in *PHD3* in primary non-small-cell lung cancer (NSCLC) and normal lung samples. (**A**) mRNA expression levels of *PHD1*, *PHD2* and *PHD3* in 60 primary NSCLC and 22 normal lung tissue samples. The horizontal lines represent the median and interquartile range. (**B**) mRNA expression levels of *PHD1*, *PHD2* and *PHD3* in 22 primary NSCLC and matched normal lung tissue samples. PHD mRNA expression levels from the same patient were combined together, each bar representing the ratio between PHD mRNA level in the primary tumour vs. the adjacent normal lung. Means of three independent experiments ± SD are shown. (**C**) PHD mRNA percentage changes in 22 primary NSCLC compared with matched normal lung tissue samples. PHD mRNA expression levels from the same patient were combined together, and each dot represents the percentage change in the expression of a specific PHD in tumour compared with normal lung samples. The horizontal lines represent median values with interquartile ranges.

The *PHD1* and *PHD2* mRNA expression in primary tumours of NSCLC patients was significantly decreased compared to normal lung tissue (both *p* < 0.0001). The median *PHD1* mRNA expression in NSCLC primary tumours was 5.24 (interquartile range 3.44–9.70), and that in normal lungs was 22.81 (interquartile range 16.83–36.19). The median *PHD2* mRNA expression in NSCLC primary tumours was 3.14 (interquartile range 1.88–4.44), and that in normal lung tissue was 6.75 (interquartile range 4.49–8.14). In contrast, we did not observe any differences in *PHD3* mRNA expression between NSCLC primary tumour and normal lung tissues (*p* = 0.256). The median *PHD3* mRNA expression in NSCLC primary tumour tissue was 11.16 (interquartile range 5.77–18.04), and that in normal lung tissue was 7.88 (interquartile range 5.06–13.52) (Figure 1A).

We also evaluated the mRNA expression of PHDs in 22 pairs of primary NSCLC tumour and adjacent normal lung tissue samples (tumour/normal lung ratio). Overall, 95.5% (21/22), 81.8% (18/22), and 45.5% (10/22) of samples displayed decreased *PHD1*, *PHD2* and *PHD3* mRNA expression, respectively, compared to adjacent normal lung tissue samples (Figure 1B). The median percentage change in PHD expression in tumour compared with paired normal lung samples was −74.9% for *PHD1*, −60.8% for *PHD2* and +13.7% for *PHD3* (Figure 1C).

**Figure 2:**
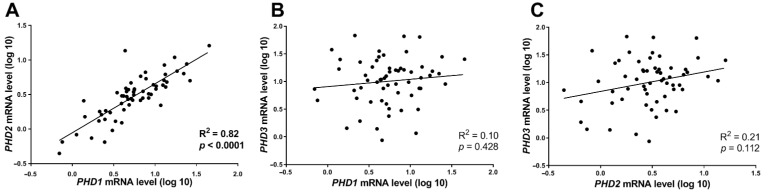
Spearman’s rank correlation coefficient analysis of associations between *PHD1*, *PHD2* and *PHD3* mRNA expression levels in 60 primary lung tumour samples. Linear regression results between (**A**) *PHD1* and *PHD2*, (**B**) *PHD1* and *PHD3* and (**C**) *PHD2* and *PHD3* mRNA levels.

The investigation of possible associations between the mRNA expression of *PHD* genes in primary tumours showed that *PHD1* and *PHD2* mRNA expression was positively correlated (*r_s_* = 0.82; *p* < 0.0001), whereas we did not observe any association between *PHD1* and *PHD3* (*r_s_* = 0.10; *p* = 0.428) or *PHD2* and *PHD3* (*r_s_* = 0.21; *p* = 0.112) mRNA expression (Figure 2).

### 3.3. Associations between PHD mRNA Levels and HIF Pathway Downstream Targets

We explored whether the different expression levels of *PHD1*, *PHD2* and *PHD3* correlated with expression levels of HIF pathway downstream genes, encoding for hypoxia-inducible factor 1α (*HIF1A*), pyruvate kinase M2 (*PKM2*) and pyruvate dehydrogenase kinase 1 (*PDK1*). Using Spearman’s rank correlation test, we found positive correlations between *HIF1A* and *PHD1* (*r_s_* = 0.56; *p* < 0.0001) and *PHD2* (*r_s_* = 0.54; *p* < 0.0001) but not with *PHD3*. Similarly, we found correlations between *PKM2* and *PHD1* (*r_s_* = 0.45; *p* = 0.0004) and *PHD2* (*r_s_* = 0.41; *p* = 0.0012) but not *PHD3*. We also found correlations between *PDK1* and *PHD1* (*r_s_* = 0.50; *p* < 0.0001) and *PHD2* (*r_s_* = 0.54; *p* < 0.0001) and *PHD3* (*r_s_* = 0.50; *p* < 0.0001) (Table 2).

### 3.4. Association between PHD mRNA Levels and Patient Characteristics

Low *PHD1* mRNA expression was associated with a higher stage of disease (*p* = 0.0089), larger tumour size (*p* = 0.0002) and squamous cell carcinoma histology (*p* = 0.0065). Similarly, low *PHD2* mRNA expression was associated with larger tumour size (*p* = 0.0026) and squamous cell carcinoma histology (*p* = 0.0050). A possible trend towards a lower expression of *PHD2* in stage II + III vs. stage I disease was observed (*p* = 0.0890). We found no correlation between *PHD3* and any of the studied clinicopathological characteristics (Table 3).

### 3.5. Association between PHD mRNA Levels and Survival

**Figure 3:**
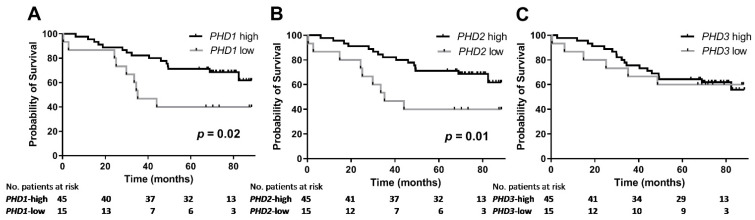
Kaplan–Meier survival curves for overall survival after surgery according to (**A**) *PHD1*, (**B**) *PHD2* and (**C**) *PHD3* mRNA expression levels in the primary tumours of 60 patients with surgically resected non-small-cell lung cancer (NSCLC). The plots show survival rates for patients with low *PHD1*/*PHD2*/*PHD3* expression (grey line) vs. patients with high *PHD1/PHD2/PHD3* expression (black line).

Patients with low *PHD1* mRNA expression had a shorter median overall survival (OS) than those with high *PHD1* mRNA expression (35.2 vs. >88.5 months; *p* = 0.02). Similarly, patients with low *PHD2* mRNA expression had a shorter median OS than patients with high *PHD2* mRNA expression (35.2 vs. >88.5 months; *p* = 0.01). No significant association was found between *PHD3* mRNA levels and OS (Figure 3). Multivariate Cox proportional hazards regression analysis adjusting for PS, pTNM stage, tumour size and nodular involvement revealed that lower *PHD1* mRNA expression in the primary tumour had borderline significance for shorter OS (HR = 0.908; 95% CI: 0.822–1.004; *p* = 0.06) (Table 4).

## 4. Discussion

Tumour hypoxia correlates with aggressive tumour phenotypes and poor prognosis in several cancer types, including lung cancer [28,29,30]. PHDs function as cellular oxygen sensors, negatively regulating the protein stability of HIF transcription factors. The HIF signalling cascade regulates the effects of hypoxia, including blood vessels formation, tumour cell invasion, and formation of metastasis. The expression of PHDs is altered in many human cancer types, but the exact mechanism of PHD deregulation has not yet been fully explained [17]. In this study, we evaluated *PHD1*, *PHD2* and *PHD3* mRNA expression in 60 NSCLC tumours and compared it to that in normal lungs and evaluated the prognostic significance of these differences for distinguishing the survival of NSCLC patients treated with radical surgery.

The results of our study showed that *PHD1* and *PHD2* mRNA expression was significantly decreased in NSCLC compared to normal lung samples. In line with this result, we also observed that decreased *PHD1* and *PHD2* were associated with larger tumour size, higher disease stage and squamous cell carcinoma histology. Similar results were also observed in a study by Giatromanolaki et al., who reported strong expression of PHD1, PHD2 and PHD3 proteins in normal bronchial epithelium and glands, whereas less than 50% of NSCLC cases were strongly positive for PHD 1, PHD2 and PHD3 [21]. The results of our study and the study of Giatromanolaki et al. contradict the results of Chen et al., who reported significantly higher expression of *PHD1*, *PHD2* and *PHD3* mRNA levels in NSCLC tissues compared with paired adjacent normal lung tissues. In contrast, the same study also reported that low *PHD3* was associated with high tumour stage and poor differentiation of tumours, suggesting that the loss of *PHD3* contributes to NSCLC invasion [22]. In another lung cancer study, Chu et al. showed that the expression of PHD3 protein was significantly higher in NSCLC tumour than in para-cancerous and normal lung tissues and positively correlated with lymph node metastasis and microvessel density [31]. On the other hand, analysis of published human cancer gene expression datasets from 14 common cancer types, including lung adenocarcinoma, revealed that *PHD2* expression was significantly decreased in tumour compared to normal tissues [32]. To complement tumour tissue studies, data from NSCLC cellular models show that *PHD1* overexpression blocks A549 lung cancer cell proliferation and tumour growth in lung cancer cell xenografts [33]. Similarly, *PHD2* was reduced in a number of cancer cell lines, and the loss of PHD2 increased tumour growth in colon carcinoma cell line xenografts.

We also found that decreased *PHD1* and *PHD2* mRNA levels were associated with squamous cell carcinoma histology. A possible explanation could be the different distribution of pTNM stages between the two histological types. The majority of patients with adenocarcinoma were in stage I (24/35; 69%), and the majority of patients with squamous cell carcinoma were in stage II or III (15/20; 75%), while only 25% were stage I. This difference in pTNM distribution is supported by published data, which indicate that, in patients with surgically resected NSCLC, squamous cell carcinoma patients have a higher stage (higher T and N status) than adenocarcinoma patients. However, squamous histology alone was not found to be an independent predictor of survival [34]. Since our results showed that reduced *PHD1* and *PHD2* were associated with larger tumours and higher tumour stage, this result could be mostly a reflection of the different stages within subgroups rather than differences in biology between histologies.

A strong correlation between *PHD1* and *PHD2* mRNA expression was found in our study, but there were no significant correlations between *PHD3* mRNA expression and the expression of the other tested PHDs. This observation suggests that the expression of *PHD1* and *PHD2* is interrelated. A possible explanation for our result could be that full-length *PHD1* and *PHD2* have more than 400 (407 and 426 in humans) amino acid residues and share a well-conserved hydroxylase domain in their C-terminal halves, whereas the N-terminal halves are more divergent with poorly characterized functions. On the contrary, the much shorter *PHD3* (239 amino acid residues in humans) contains a hydroxylase domain but only a short segment of the divergent N-terminal sequence [16].

To further explore how decreased *PHD 1* and *PHD2* mRNA levels affect other HIF pathway downstream targets, we evaluated correlations between all three studied PHD isoforms and genes encoding for HIF1α, PKM2 and PDK1. The active HIF complex is composed of one oxygen-regulated α-subunit (HIF-α) that is regulated and one constitutively expressed β-subunit. There are three HIF-α family members (HIF-1α, -2α and -3α). The pyruvate kinase M2 (PKM2) is a glycolytic enzyme induced by HIF1α, which have roles in the development, progression, and metabolism of cancer. PKM2 interacts directly with the HIF-1α subunit and stimulates HIF-1 transcriptional activity [35,36]. In addition to promoting glycolysis, HIF-1 also inhibits the oxidation glucose through the tricarboxylic acid cycle (TCA) in the mitochondria by upregulating the gene which encodes for pyruvate dehydrogenase kinase 1 (PDK1), thus inactivating pyruvate dehydrogenase (PDH), which converts pyruvate to acetyl-CoA [15,37]. Our results showed positive correlations between *PHD1* and *PHD2* and all three studied HIF downstream targets, which further supports our results that decreased *PHD1* and *PHD2* are associated with the development of NSCLC.

Our results also showed that low mRNA expression of *PHD1* and *PHD2*, but not *PHD3*, was associated with shorter OS of patients. In addition, *PHD1* showed borderline independent prognostic value in the multivariate analysis. *PHD1* was moderately well correlated with tumour size and hence also with tumour stage, as these measures represent similar information. As a consequence, other variables were eliminated in the multivariate analysis. To date, a limited number of studies have assessed survival outcomes in relation to the expression of PHDs, including one in NSCLC [38,39,40,41]. Our results are the opposite of the results of Andersen et al., who showed that positive PHD1 and PHD2 protein expression are independent negative prognostic factors in NSCLC [38]. PHDs have been proposed as both tumour suppressors and drivers of tumorigenesis [17]. Our results support the thesis that PHDs function as tumour suppressors, their expression being reduced in tumours, which correlates with poor prognosis of patients, whereas the results of Andersen et al. support the hypothesis that PHDs have pro tumour activity. In other cancer types, high nuclear PHD1 or PHD3 protein expression was associated with poorer survival of patients with pancreatic endocrine tumours [39]. Conversely, in gastric cancer, patients with negative PHD2 protein expression had significantly shortened survival in comparison with PHD2-positive patients [40]. Additionally, in hepatocellular carcinoma, patients with reduced *PHD3* mRNA expression had shorter survival (DFS and OS) and higher disease recurrence rates [41]. These controversial data indicate that further exploration of the role of PHDs in cancer progression is needed. It also appears that PHDs are differentially dysregulated in different types of cancer. As also stated in Jokilehto and Jaakkola’s review article, ‘‘given the uncertainties in specific PHD function, their role in cancer is inconclusive at the best” [17].

A possible explanation for the discrepancy between different studies evaluating the expression profiles and prognostic significance of PHD proteins in (lung) cancer may be the different methodological approaches for PHD measurements. Most of the already published studies used immunohistochemistry (IHC) to determine PHD protein expression in lung cancer tissue [21,31,42], and only one study was based on gene expression analysis with RT-qPCR. The main advantage of RT-qPCR over IHC is its ability to quantify gene expression compared to the semiquantitative protein expression scoring. There are a limited number of studies directly comparing mRNA and protein expression of PHDs. In cell lines, the protein levels of PHD2 and PHD3 seem to correlate well with the mRNA levels, but PHD1 protein levels were lower than one would expect from the mRNA levels [43]. The expression of PHDs may also vary because of the different antibodies and primers used, and different cut-offs to categorize patients with high/low expression of PHDs were used in different studies. To eliminate these sources of variability to some extent, we used expression assays that target all different transcripts/isoform if present and used continuous values of *PHD* mRNA expression when possible (e.g., Mann–Whitney, Cox regression). In addition, there is evidence that PHD expression is also regulated at the transcriptional and posttranslational levels by proteasomal destruction and protein interactions. In future studies, it would be necessary to directly compare mRNA and protein expression levels of different PHD isoforms in primary NSCLC tumours, taking into account different IHC antibody clones that are currently available. A possible reason for the differences in results between different studies might also be intratumoural heterogeneity [44]. This confounder could partially be overcome with tissue sampling from several tumour sites. However, our study did not use samples from various tumour sites, which is one of the limitations of our study. Additionally, the relatively small sample size may make this study susceptible to unknown bias. A possible effect of PHDs downregulation on the HIF pathway was shown with a significant correlation to *HIF1A*, *PKM2* and *PDK1* mRNA levels; however, these are only indirect pieces of evidence regarding mechanism exploration. Therefore, additional mechanistic studies, potentially involving PHD knock-out mouse models, are necessary to evaluate these associations.

## 5. Conclusions

In the present study, we showed that the mRNA expression of prolyl hydroxylase domain proteins, specifically *PHD1* and *PHD2*, in NSCLC primary tumours was decreased, which correlated with the expression of HIF pathway downstream genes, with larger tumour size and poor prognosis of patients. This result suggests that loss of *PHD1* and *PHD2* expression is associated with the development and progression of NSCLC. Larger studies are needed to further evaluate *PHD1* as a marker of unfavourable prognosis in surgically resected NSCLC. Identification of hypoxic markers in tumours could improve the efficacy of current cancer therapies, including immunotherapy.

## Figures and Tables

**Table 1:** Clinical and pathological characteristics of 60 non-small-cell lung cancer (NSCLC) patients.

Characteristics		NSCLC *N* (%)
*N*° of patients		60
Age in years: median (range)		61 (42–79)
Sex		
	Male	33 (55.0)
	Female	27 (45.0)
Smoking history		
	Yes	51 (85.0)
	No	9 (15.0)
Histology		
	Adenocarcinoma	35 (58.4)
	Squamous cell carcinoma	20 (33.3)
	Other histotype	5 (8.3)
pTNM stage ^a^		
	I	31 (51.6)
	II	16 (26.7)
	III	13 (21.7)
Tumour size		
	pT1 (<3 cm)	24
	pT2 (3–7 cm)	28
	pT3 (>7 cm)	8
Lymph node involvement ^Ϯ^		
	pN0	39
	pN1	7
	pN2	13
Performance status ^b^		
	0	21 (35.0)
	1	37 (61.7)
	≥2	2 (3.3)

*N*°: number of patients; NSCLC: non-small-cell lung cancer; ^a^ AJCC cancer staging 7th edition [27]. ^b^ East Cooperative Oncology Group performance status. ^Ϯ^ Lymph node dissection was not done in one patient.

**Table 2:** Spearman’s rank correlation coefficient analysis of associations between *PHD1*, *PHD2* and *PHD3* mRNA levels and HIF pathway downstream regulators *HIF1A*, *PKM2* and *PDK1* in 60 primary lung tumour samples.

HIF Target	*PHD1*	*PHD2*	*PHD3*
*HIF1A*	*r_s_* = 0.56 (*p* < 0.0001)	*r_s_* = 0.54 (*p* < 0.0001)	*r_s_* = 0.037 (*p* = 0.78)
*PKM2*	*r_s_* = 0.45 (*p* = 0.0004)	*r_s_* = 0.41 (*p* = 0.0012)	*r_s_* = 0.24 (*p* = 0.06)
*PDK1*	*r_s_* = 0.50 (*p* < 0.0001)	*r_s_* = 0.54 (*p* < 0.0001)	*r_s_* = 0.50 (*p* < 0.0001)

**Table 3:** Correlations between PHD mRNA expression level and clinicopathological characteristics of 60 surgically resected non-small-cell lung cancer (NSCLC) patients.

Parameter		*N*° of ps	*PHD1* mRNA Level	*p*	*PHD2* mRNA Level	*p*	*PHD3* mRNA Level	*p*
Age								
	<60	29	5.60 (3.40–9.88)	0.7561	3.49 (2.05–4.51)	0.4159	10.85 (7.40–17.79)	0.4506
	≥60	31	5.13 (2.47–10.30)		2.95 (1.51–4.80)		11.47 (3.93–18.32)	
Sex								
	Male	33	5.35 (2.46–9.88)	0.6030	2.95 (1.65–4.35)	0.5132	9.72 (5.93–17.77)	0.6665
	Female	27	5.11 (3.95–11.69)		3.49 (1.90–4.80)		12.53 (4.98–18.32)	
Smoking history								
	Yes	51	5.11 (3.04–8.37)	0.1654	2.95 (1.79–4.33)	0.1020	10.78 (4.98–18.29)	0.7719
	No	9	9.53 (4.29–13.64)		4.07 (3.34–5.49)		11.65 (8.60–17.81)	
Histology ^a^								
	Adenocarcinoma	35	6.70 (4.51–12.41)	0.0065	3.68 (2.76–5.35)	0.0050	10.78 (4.27–16.22)	0.0624
	Squamous cell carcinoma	20	3.71 (1.59–6.33)		2.23 (1.25–3.63)		15.44 (8.69–25.22)	
pTNM stage								
	I	31	7.10 (4.20–13.29)	0.0089	3.52 (2.23–5.35)	0.0890	12.09 (3.19–18.48)	0.5303
	II + III	29	4.43 (2.36–7.17)		3.17 (2.21–4.66)		10.78 (7.40–18.30)	
Tumour size								
	pT1	24	8.36 (4.78–16.36)	0.0002	3.98 (2.99–6.31)	0.0026	12.87 (4.28–21.40)	0.5845
	pT2-3	36	4.27 (2.32–6.68)		2.66 (1.53–3.77)		9.72 (5.77–17.29)	
Nodular involvement ^Ϯ^								
	Yes	20	4.38 (2.61–6.68)	0.0795	2.66 (1.32–3.87)	0.2166	10.82 (7.51–18.04)	0.5620
	No	39	5.53 (3.96–11.69)		3.17 (2.21–4.66)		11.47 (4.48–24.00)	

*N*°: number of patients; ^a^ Five patients with other histotypes were excluded from the analysis. The data are presented as the median with interquartile range. ^Ϯ^ Lymph node dissection was not done in one patient.

**Table 4:** Univariate and multivariate Cox proportional hazards regression model analyses of survival.

Parameter	Overall Survival	
	UV *p*-Value HR (95% CI)	MV *p*-Value HR (95% CI)
*PHD1* mRNA level	0.060 0.908 (0.822–1.004)	0.060 0.908 (0.822–1.004)
*PHD2* mRNA level	0.481 0.940 (0.792–1.116)	n/i
*PHD3* mRNA level	0.458 0.988 (0.958–1.020)	n/i
Age (>60 vs. ≤60)	0.866 0.933 (0.417–2.086)	n/i
Sex (M vs. F)	0.399 1.428 (0.624–3.265)	n/i
Smoking history (Yes vs. No)	0.654 0.758 (0.225–2.548)	n/i
Histology (AC vs. SCC)	0.565 0.778 (0.332–1.826)	n/i
PS ^a^ (≥2 vs. <2)	0.217 0.558 (0.221–1.408)	Eliminated ^b^
pTNM stage (I vs. II vs. III) I vs. II II vs. III	0.102 0.364 (0.140–0.944) 0.439 (0.149–1.295)	Eliminated ^b^
Tumour size (pT1 vs. pT2-3)	0.231 0.584 (0.242–1.409)	Eliminated ^b^
Nodular involvement (Yes vs. No)	0.051 0.439 (0.192–1.003)	Eliminated ^b^

*N*: number of patients; CI: confidence interval; HR: hazard ratio; UV: univariate analysis; MV: multivariate analysis; SCC: squamous cell carcinoma; AC: adenocarcinoma; n/i: not included; ^a^ East Cooperative Oncology Group performance status; ^b^ Backward conditional stepwise regression eliminated this variable from the model.

## Data Availability

Data is contained within the article.
